# Construction of a high-density genetic map and localization of grazing-tolerant QTLs in *Medicago falcata* L.

**DOI:** 10.3389/fpls.2022.985603

**Published:** 2022-10-03

**Authors:** Xinyue Zhou, Xiaojie Li, Xiaoming Zhang, Dabao Yin, Junjie Wang, Yan Zhao

**Affiliations:** Key Laboratory of Grassland Resources (IMAU), Key Laboratory of Forage Cultivation, Processing and High Efficient Utilization, College of Grassland, Resource and Environmental Science, Ministry of Education, Ministry of Agriculture, Inner Mongolia Agricultural University, Hohhot, China

**Keywords:** *Medicago falcata* L., grazing tolerance, genetic linkage mapping, QTLs, high-density

## Abstract

**Background:**

Using genomic DNA from 79 F1 plants resulted from a crossing between parents with strong and weak grazing tolerance in *Medicago falcata* L., we generated an *EcoRI* restriction site-associated DNA (RAD) sequencing library. After sequencing and assembly, a high-density genetic map with high-quality SNP markers was constructed, with a total length of 1312.238 cM and an average density of 0.844 SNP/cM.

**Methods:**

The phenotypic traits of 79 F1 families were observed and the QTLS of 6 traits were analyzed by interval mapping.

**Results:**

Sixty three QTLs were identified for seven traits with LOD values from 3 to 6 and the contribution rates from 15% to 30%. Among the 63 QTLs, 17 were for natural shoot height, 12 for rhizome Length, 10 for Shoot canopy diameter, 9 for Basal plant diameter, 6 for stem number, 5 for absolute shoot height, and 4 for rhizome width. These QTLs were concentrated on LG2, LG4, LG5, LG7, and LG8. LG6 had only 6 QTLs. According to the results of QTL mapping, comparison of reference genomes, and functional annotation, 10 candidate genes that may be related to grazing tolerance were screened. qRT-PCR analysis showed that two candidate genes (LOC11412291 and LOC11440209) may be the key genes related to grazing tolerance of *M. falcata.*

**Conclusion:**

The identified trait-associated QTLs and candidate genes in this study will provide a solid foundation for future molecular breeding for enhanced grazing-tolerance in *M. falcata.*

## Introduction

*Medicago falcata* L. is a perennial legume in the *Medicago* genus (Wang et al., 2006, 2016). Distributed mainly in alpine regions such as Russia, Mongolia, and China’s East Central Inner Mongolia and Xinjiang. *Medicago falcata* has a strong tolerance to cold, drought, and grazing, and can grow in marginal soil with wide adaptability (Shi et al., 2019; Zhou et al., 2021). Because of these application values, *M. falcata* is widely used for grassland improvement, artificial range setup, and sand prevention (Popp et al., 2000). Improving grazing tolerance in cultivated alfalfa is a common goal worldwide in alfalfa breeding. In this regard, the values of *M. falcata* are well accepted for alfalfa breeding (Pecetti et al., 2008). In *M. falcata*, many traits, such as rhizome length, stem number, and shoot canopy diameter, are quantitative traits. It is practical to locate these traits in the *M. falcata* genome by using QTL analysis (McCord et al., 2014). The development and application of single nucleotide polymorphism (SNP) and the emergence of restriction site-associated DNA sequencing (RAD-Seq) technology pave the way for rapid development of SNPs and subsequent QTL analysis in non-model species, such as *M. falcata* (Li and Brummer, 2012; Kang et al., 2014). RAD-Seq is a new, yet powerful technology, which allows quick identification of millions of SNPs in a mapping population at low cost (Kumar et al., 2009; José et al., 2017; Yermekbayev et al., 2020). With RAD-Seq, we are able to generate a large number of SNPs, construct a genetic linkage map, and perform QTL analysis in *M. falcata*. Yin (2021) studied genetic diversity of *Elymus dahuricus* was analyzed by RAD-Seq sequencing. The results showed that the phenotypic clustering results were consistent with RAD sequencing results for about 50% of the materials within two years, indicating that phenotypic classification results and molecular sequencing results were mutually confirmed and well matched. For example, (Zhang et al., 2020) used single nucleotide polymorphism (SNP) markers to construct a high-density linkage map of alfalfa. QTL mapping for yield-related traits was carried out. Cui (2020) used 460 SNP markers to construct the genetic linkage map of alfalfa and *M. falcata*, and conducted QTL mapping for alfalfa agronomic traits. Liu (2012) constructed a genetic linkage map of tetraploid alfalfa using 51 RAPD markers and mapped QTL for 16 important agronomic traits. Liu (2017) constructed alfalfa genetic linkage map by using 176 SSR polymorphism markers and 960 SNP markers, and conducted QTL mapping for 19 related traits, such as alfalfa yield. Up to date, numerous studies have demonstrated the phenotypic features related to grazing tolerance, established the evaluation methodologies and metrics for grazing tolerance in *M. falcata*, and proven the unique advantages of *M. falcata* in the improvement of grazing tolerance in cultivated alfalfa. Wang et al. (2013) proved the reliability of optimal sequence analysis in *M. falcata* grazing tolerance, and through morphological index analysis showed that in the Hulunbuir native *M. falcata*, plant individuals with large projection area, great plant height, significant plants diameters stem number, long root depth and significant root diameters had strong grazing tolerance. Wang (2015) studied the grazing tolerance in Hulunbuir native *M. falcata*, cloned and analyzed some grazing tolerance-related genes (Jiang et al., 2022). Based on RAD-seq, the genetic linkage map and QTL mapping of *Medicago sativa* L. flowering stage traits were constructed, and 7 candidate genes related to flowering stage were screened out. However, there are no reports on the construction of genetic linkage map of *M. falcata*, QTL studies on grazing tolerance traits of *M. falcata,* and candidate gene screening (Luciano et al., 2021).

In this study, we chose two *M. falcata* parents with contrasting grazing tolerance, crossed the two parents, and generated a mapping population. Using RAD-seq technique, SNPS were identified, the first genetic linkage map was constructed, and QTL sites related to grazing tolerance were analyzed. Ten candidate genes related to grazing tolerance were screened out, and two of them were presumed to be key genes. The linkage map constructed and the QTL candidate genes identified for grazing tolerance will provide valuable information for future molecular mechanism studies and lay a solid foundation for improving grazing tolerance of alfalfa.

## Materials and methods

### Materials and population generation

Two native *M. falcata* genotypes (MF200401 and MF200402) from Hulunbuir, Inner Mongolia, China, were chosen as the parents for genetic crossing, and they were tetraploid plants. MF200401, which has high grazing tolerance, was used as the maternal parent, whereas MF200402, which has low grazing tolerance, was used as the paternal parent. Individual plants with contrasting grazing tolerance phenotypes were manually pollinated to generate an F1 population of 79 plants. From 2016 to 2018, we observed the agronomic traits related to grazing tolerance of *M. falcata* F1 population at grassland station of Hulunbuir Ewenki Autonomous Banner (119°07′ E latitude 49°01′ N latitude) and experimental base of Inner Mongolia Agricultural University, and focused on the identification and evaluation of grazing tolerance.

### Trait definition and analysis

In this study, we focused on the following important traits: shoot height, basal plant diameter, stem number, shoot canopy diameter, rhizome length, and rhizome width. Each trait was measured with the following description:

Shoot height (cm): We measured the natural height and the absolute height. Natural height was measured as the height of shoot from the ground to the highest point of the shoot under natural growing status. Absolute height was measured as the height of shoot from the ground to the highest point of the shoot when the shoot was pulled and stretched straight.
Shoot basal diameter (cm): The diameter of the cluster below the first node was measured.
Stem number: Total number of shoots in a plant cluster.
Shoot canopy diameter (cm): The shoot canopy diameter was approximated by measuring the diameter of the shadow cast by the shoot canopy on the ground when the sun was on the top of the plant at noon.
Rhizome length (cm) and rhizome width(cm): The length and the diameter of the crown of each cluster of plants were measured.

### DNA extraction

From the crossing population, 79 individuals were selected randomly to construct the genetic map. Genomic DNA of the 79 F1 individuals and two parents, MF200401 and MF200402, was extracted from young leaves using the Plant Genomic DNA Extraction Kit (DP305, TianGen, Beijing, China) following the manufacturer’s instructions.

### RAD library construction and sequencing

About 1 μg of genomic DNA was digested with *EcoRI*, followed by ligation of Solexa P1 Adapter (common adapter with EcoRI end), fragmentation, gel recovery of 300–700 bp fragments, end-filling of A, ligation of Solexa P2 Adapter (adapter with barcode). The individual samples were pooled together, purified, and PCR amplified with P1 and P2 primers for 18 cycles. The 300–700-bp amplicons were further gel purified and the quality was checked using Agilent 2100 Bioanalyzer and then sequenced using the Illumina HiSeq 2500 platform at BGI (Shenzhen, China).

### Sequencing data analysis

In order to ensure the accuracy of subsequent information analysis, the original sequence was quality-filtered and compared to the reference genome for analysis by BWA software. Sequence data were analyzed using customized Perl scripts from BGI-Shenzhen (Shenzhen, China). Raw reads were cleaned up by removing the adapters, index sequences, and low-quality reads. RAD markers were developed using the clean data. SNPs markers were examined using the GATK^1^ program. Reads from each individual were clustered into tag reads by sequence similarity (allowing five mismatches, at most, between any two reads within each tag reads cluster) and clusters with <3 or >100 reads were discarded. All the SNPs had total support reads ≥ 5, and for heterozygous SNPs, the inferior base depth was ≥3.

### SNP detection and map construction

All SNPs markers used for genetic linkage map construction were filtered using the following criteria: (1) ratio of confidence levels to quality depth ≥ 2; (2) *p*-values from the Fisher’s exact test ≤ 60; (3) RMS mapping quality values ≥ 40; (4) all markers were tested by Chi-square test (*p* < 0.01).

Genetic linkage maps were generated using JoinMap version 4.1. A logarithm of the odds (LOD) score between 2 and 20 was set to cluster linkage groups. The regression mapping was used as the mapping algorithm, and the genetic distances were calculated based on Kosambi’s mapping function. All SNPs were clustered on 8 linkage groups (LGs). The location and distance between each SNP were calculated based on multipoint analysis.

### QTLs analysis

QTL analysis was performed using MapQTL version 6.0 software based on the parental maps and phenotype data from 79 individual plants. QTLs were detected using interval mapping initially, and the mapping algorithm was a mixed model. Then multiple QTL mapping (MQM) was performed to detect additional QTLs that might be masked by the major QTLs. After a 1,000 permutation test, a LOD threshold of 3 was set to find significant QTLs at the 95% confidence level. The ranges above the LOD threshold of 3 were identified as QTL intervals. Markers located at or flanked with the peak LOD value of a QTL were recognized as QTL-associated markers.

### Screening and analysis of candidate genes

*Medicago falcata* L. CV. Hulunbuir was selected as plant material to detect the expression of candidate genes. Choose the particle satiated *M. falcata*, with sandpaper, break hard real-time, in Petri dish culture to sprout, the germination of seeds in sterile culture in the soil and put to cultivate in artificial climate chamber, during the cultivation for long sunshine condition (16 h light/8 h) of the dark, day/night temperature 18°C to 26°C, well ventilated, and regular watering. When growth to the flowering period, choose three plants that grow better and the peak of flowering period simulated cutting processing, *M. falcata* cutting stubble height is 15 cm, respectively dealing with 3, 5, and 7 days, each point in time selecting a suitable amount of leaf and stem tissue in 2 ml centrifuge tube, and one not to cut processing plant as a control, sampling at the same time, the liquid nitrogen frozen. Total RNA was extracted according to the FastPure Plant Total RNA Isolation Kit (Vazyme, China), and biological replicates were performed three times. The quality of RNA was determined by electrophoresis of a 1% (w/v), agarose gel. First-strand cDNA was synthesized from 2 μg total RNA using the TransScript First-Strand cDNA Synthesis SuperMix Kit (TransGen, China). qRT-PCR was performed by SYBR green Super Mix and CFX96 Real-Time PCR Detection System (Bio-Rad, Hercules, CA, United States). Gene-specifc primers used for qRT-PCR were designed using Oligo 7.0. The expression level of *MfActin* was used as the internal control, the relative expression levels of candidate genes were calculated according to the 2^−ΔΔCT^ method, and three independent biological replicates were used for each sample.

## Results

### Establish mapping groups

**Figure 1:**
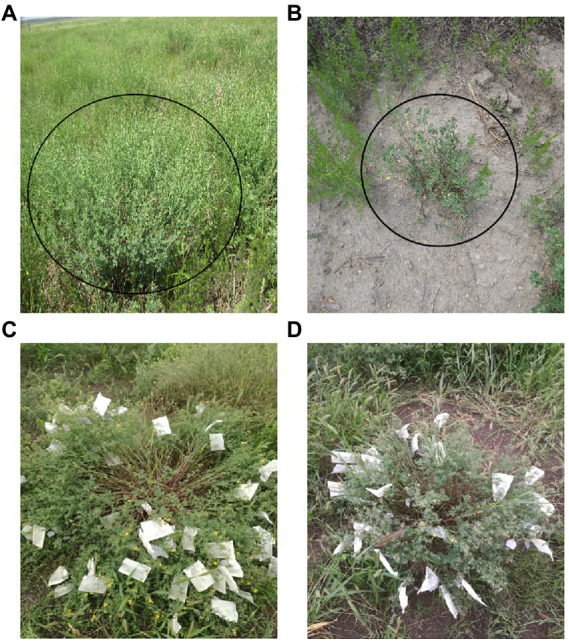
**(A)** Plants with high grazing tolerance were selected under continuous high-intensity grazing in Ewenki Autonomous Banner (119° 07′ E Latitude 49° 01′ N Latitude), Hulunbuir, China, and named (MF200401); **(B)** Plants with low grazing tolerance were selected under continuous high-intensity grazing in Ewenki Autonomous Banner (119° 07′E Latitude 49° 01/N Latitude), Hulunbuir, China, and named (MF200402); **(C)** Orthologous population; hybrid parent 1; 79 F1 populations obtained by crossing grazing-tolerant material (MF200401) with poorly grazing-tolerant material (MF200402); **(D)** Reverse cross population; hybrid parent 2; 54 F1 populations obtained by crossing grazing-tolerant material (MF200401) with poorly grazing-tolerant material (MF200402).

In this study, two alfalfa populations with extreme differences in grazing tolerance traits were selected as materials, and the parents with the greatest differences in important grazing tolerance-related traits such as regeneration rate, number of branches, and root neck depth into the soil were selected for intra-species one-to-one single sexual crosses to obtain two F1 populations, which could be asexually propagated and could be used as permanent mapping populations, and a population with 79 single plants was selected as the mapping population after hybrid testing and identification (Figure 1).

### Analysis of variance of morphological index data observed after grazing

The morphological and physiological characteristics related to grazing tolerance of *M. falcata* were studied systematically. Five phenotypic traits directly affecting the formation of grazing tolerance of *M. falcata* and two phenotypic traits indirectly involved in the formation of grazing tolerance were identified, which revealed the morphological mechanism and physiological basis of grazing tolerance of *M. falcata*. Two genotypic population materials with significant differences in grazing tolerance were identified and screened (one was MF200401 material with high grazing tolerance, and one is MF200402 material with low grazing resistance; Table 1).

**Table 1:** Analysis of variance of phenotypic traits observed in F1 population of two sites in 3 years was conducted.

	Non-grazing plant	The grazing resistance is high	The grazing resistance is low
Plant individuals with large projection area(cm^2^)	5897.66a	3754.98a	1600.87a
Basal plant diameter(cm)	5.71c	11.73a	8.29b
Absolute shoot height(cm)	79.44a	63.98b	47.76a
Natural shoot height (cm)	77.20a	60.90b	44.36c
Stem number(a)	72.30a	62.14b	22.43c
Rhizome length(cm)	4.56b	4.19a	2.45c
Rhizome width(cm)	2.01a	2.84a	2.05a

There were significant differences between the morphological indexes of high grazing tolerance and low grazing tolerance, which indicated that continuous grazing had significant effects on the morphological characteristics of plants, and was beneficial to the expression of differences in grazing tolerance. Different letters indicate significant difference (*P* < 0.05).

### Polymorphism selection

In this study, a total of 1,412,614 high-quality polymorphic SNP loci were screened based on the RAD data of the parents. Based on the SNP detection results, the polymorphic SNPs between parents were screened (Table 2). For the F1 population, heterozygous loci with polymorphisms between parents (lm × ll, nn × np, ab × cd, ef × eg, hk × hk types) were screened (Table 3). Filter out loci with missing parental information. The marker loci with >10% deletion rate in the offspring population were filtered out, i.e., for single polymorphic loci, at least 90% of the samples have a definite genotype. After filtering to obtain parental polymorphic loci meeting the filtering 5,191 parental polymorphic loci were filtered, and the results were imported into JoinMap 4.1 software for further.

**Table 2:** The results of mutation detection and analysis by high-throughput sequencing showed that summary of SNPs in individual samples.

Sample_ID	Total	Homo	Hete	Homo_rate(%)	Hete_rate(%)
H01	629,785	570,191	59,594	90.54	9.46
H02	524,816	494,067	30,749	94.14	5.86
H04	562,791	523,745	39,046	93.06	6.94
H10	658,919	581,161	77,758	88.2	11.8
H11	671,246	590,520	80,726	87.97	12.03
H12	639,810	575,487	64,323	89.95	10.05
H13	611,792	558,902	52,890	91.35	8.65
H14	610,369	558,251	52,118	91.46	8.54
H15	590,379	544,253	46,126	92.19	7.81
H16	599,237	549,814	49,423	91.75	8.25
H17	560,188	521,924	38,264	93.17	6.83
H18	346,826	336,690	10,136	97.08	2.92
H19	628,386	569,464	58,922	90.62	9.38
H20	709,345	578,355	130,990	81.53	18.47
H21	706,928	583,209	123,719	82.5	17.5
H22	716,673	556,186	160,487	77.61	22.39
H24	538,661	504,281	34,380	93.62	6.38
H25	482,521	456,552	25,969	94.62	5.38
H26	603,447	553,801	49,646	91.77	8.23
H27	542,397	506,108	36,289	93.31	6.69
H29	528,781	495,772	33,009	93.76	6.24
H30	639,210	574,955	64,255	89.95	10.05
H31	699,137	579,127	120,010	82.83	17.17
H32	683,180	583,133	100,047	85.36	14.64
H33	467,812	443,643	24,169	94.83	5.17
H34	374,120	361,015	13,105	96.5	3.5
H36	437,006	417,590	19,416	95.56	4.44
H37	544,192	507,617	36,575	93.28	6.72
H38	451,103	429,651	21,452	95.24	4.76
H39	697,647	581,017	116,630	83.28	16.72
H43	543,351	507,551	35,800	93.41	6.59
H44	643,833	579,401	64,432	89.99	10.01
H45	538,746	503,634	35,112	93.48	6.52
H47	563,069	519,805	43,264	92.32	7.68
H48	508,906	477,803	31,103	93.89	6.11
H49	279,432	273,067	6,365	97.72	2.28
H50	359,469	347,522	11,947	96.68	3.32
H51	569,924	526,554	43,370	92.39	7.61
H52	716,452	547,454	168,998	76.41	23.59
H53	640,006	574,547	65,459	89.77	10.23
H54	685,413	591,110	94,303	86.24	13.76
H55	579,611	535,577	44,034	92.4	7.6
H56	584,826	537,408	47,418	91.89	8.11
H58	619,020	563,143	55,877	90.97	9.03
H59	691,599	588,375	103,224	85.07	14.93
H61	706,157	578,625	127,532	81.94	18.06
H62	713,247	563,881	149,366	79.06	20.94
H63	714,794	560,315	154,479	78.39	21.61
H64	683,965	582,759	101,206	85.2	14.8
H65	618,592	558,140	60,452	90.23	9.77
H66	525,975	491,979	33,996	93.54	6.46
H67	550,167	511,867	38,300	93.04	6.96
H68	664,101	578,075	86,026	87.05	12.95
H69	595,697	544,149	51,548	91.35	8.65
H71	640,511	573,262	67,249	89.5	10.5
H72	628,274	559,643	68,631	89.08	10.92
H73	676,475	576,695	99,780	85.25	14.75
H74	581,103	529,890	51,213	91.19	8.81
H75	624,013	562,901	61,112	90.21	9.79
H76	603,548	546,388	57,160	90.53	9.47
H77	560,243	515,244	44,999	91.97	8.03
H78	639,971	566,110	73,861	88.46	11.54
H79	589,320	536,058	53,262	90.96	9.04
H80	179,512	176,698	2,814	98.43	1.57
H81	502,366	470,751	31,615	93.71	6.29
H83	505,541	473,952	31,589	93.75	6.25
H84	558,170	518,611	39,559	92.91	7.09
H85	706,562	578,636	127,926	81.89	18.11
H86	682,101	581,144	100,957	85.2	14.8
H87	700,590	581,675	118,915	83.03	16.97
H88	702,105	577,375	124,730	82.23	17.77
H89	707,803	581,006	126,797	82.09	17.91
H90	679,030	588,952	90,078	86.73	13.27
H92	696,768	584,581	112,187	83.9	16.1
H93	712,176	565,509	146,667	79.41	20.59
H94	677,064	587,480	89,584	86.77	13.23
H95	706,589	576,568	130,021	81.6	18.4
H96	592,612	540,033	52,579	91.13	8.87
H97	475,846	450,240	25,606	94.62	5.38
P1	696,915	548,925	147,990	78.76	21.24
P2	715,699	486,454	229,245	67.97	32.03

Homo is the number of homozygous SNPs. Hete is the number of heterozygous SNPs. Homo rate and Hete rate are the rate of homozygous and heterozygous SNPs in total SNPS, respectively. It was noted that the Homo rate and Hete rate in individual F1 samples varied greatly. The lowest Hete rate (samples H80) was only 1.57%, whereas the highest (sample H52) was 23.59%.

**Table 3:** Type of markers.

Parent genotype	Marker type explanation	F1	F2/BC/DH/RIL	Progeny genotypes		
				F1	F2	DH/RIL
aa × bb	Both parents have different homozygous loci		√		aa, ab, bb	aa, bb
lm × ll	Parent 1 is heterozygous whereas parent 2 is homozygous	√		ll, lm		
nn × np	Parent 1 is homozygous whereas parent 2 is heterozygous	√		nn, np		
ab × cd	Both parents are heterozygous (four alleles)	√		ac, ad, bc, bd		
ef × eg	Both parents are heterozygous (three alleles)	√		ee, eg, ef, fg		
hk × hk	Both parents are heterozygous (two alleles)	√		hh, hk, kk		

### Biased segregation filtering In progenies

The offspring were genotyped according to the parental marker types obtained from the screening, and the obtained markers were tested by Chi-square test (significance level α = 0.01) to remove the segregating markers (e.g., F2 population aa, ab, bb). The expected probability ratio of F2 population aa, ab, and bb genotypes is 1:2:1, and a significant deviation from this ratio is considered as a marker bias, the bias markers will affect the map construction results and QTL localization, and the majority of the literature on the treatment of biased segregation, using the Chi-square test. The threshold value for segregation was set at 0.1, and the abnormal genotypes were filtered out according to the segregating genotypes of the offspring of different populations. The results were compiled into the Joinmap4.1 input file format for genetic mapping (Table 4).

**Table 4:** Summary of polymorphic types in F1 progenies.

Gene_type	Count	Rate (%)
hkxhk	115,902	45.52
nnxnp	107,427	42.2
lmxll	30,388	11.94
efxeg	876	0.34
abxcd	2	0

The number of gene type hkxhk was 115,902, with a ratio of 45.52%. The number of gene type abxcd was 2, with a ratio of 0.

### Construction of genetic linkage map

**Figure 2:**
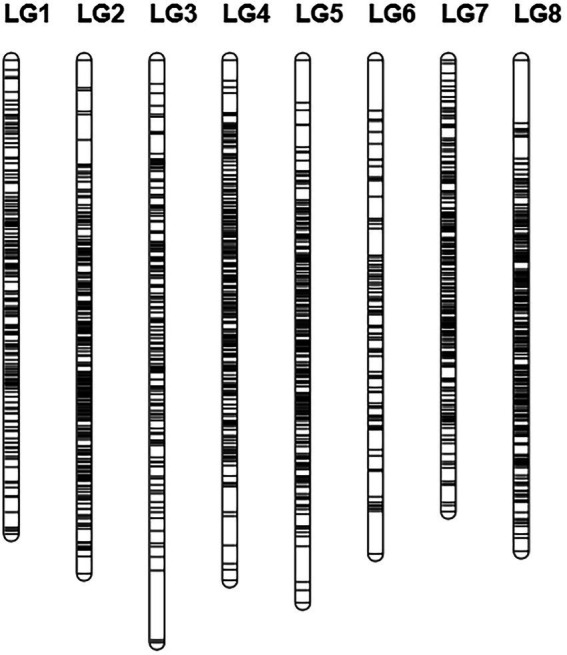
Genetic linkage map.

Based on logarithm of the odds (LOD) values (2 < LOD < 20), 1,756 SNPs were used to generate the genetic linkage map using JoinMap version 4.1. The regression mapping was used as the mapping algorithm, and the genetic distances were calculated based on Kosambi’s mapping function. These mapped SNPs were clustered in 8 linkage groups (LGs). The total length of the consensus map was 1312.238 cM with the average distance about 0.844 cM between markers. The distribution of SNPs in each LG and the genetic distances were summarized in Table 4 From the results in Table 4, LG4 and LG8 had the most SNPs, while LG6 had the least SNPs. LG8 had the smallest average genetic distance between SNPs, while LG6 had the largest average genetic distance of 1.69 cM. The largest gap between two SNPs (22.482 cM) was found in LG3. All SNPs were loaded to Joinmap4.1 and a genetic map was generated as shown in Figure 2.

### Co-linear analysis of genetic map and physical map

From the collinearity analysis results, the collinearity results of genetic map and physical map were not very good, but the trend of most markers was consistent. Marker was evenly distributed on the genome.

### Analysis of grazing tolerance QTLs

QTLs were detected using the interval mapping method in the MapQTL6 software. A total of 63 QTLs were identified for six grazing tolerance-associated traits (Table 5). Among them, 12 QTLs were related to rhizome length, which contributed for 16.9%–28.3% variance; 4 QTLs for rhizome width, contributing for 17.7%–20.3%; 10 QTLs for shoot canopy diameter, contributing for 16.7%–26%; 9 QTLs for basal plant diameter, contributing for 16.6%–27.4%; 6 QTLs for stem number, contributing for 16.5%–22.3%; 5 QTLs for absolute shoot height, contributing for 16.8–20.7%; and 17 QTLs for natural shoot height, contributing for 16.5%–24.4%. These QTLs were mainly distributed on LG2, LG4, LG5, LG7, and LG8. LG6 had the least QTLs. The LOD values were between 3 and 6 and the contribution was between 15% and 30%. There were no markers that had significantly high contribution (greater than 60%).

**Table 5:** Results of QTL analysis from MapQTL6.

Trait	LG	Marker	Genetic distance (cM)	LOD	Contribution	Variance
Rhizome length	LG1	chr01_38977492	17.791	3.96	21.6	3.5
Rhizome length	LG1	chr01_38977519	17.929	4	21.8	3.5
Rhizome length	LG1	chr01_49110666	52.667	3.33	18.5	3.7
Rhizome length	LG1	chr01_4358824	94.374	4.78	25.4	3.4
Rhizome length	LG2	chr02_33256187	91.592	3.79	20.8	3.6
Rhizome length	LG6	chr06_3,615,590	112.904	3.03	17	3.3
Rhizome length	LG3	chr03_52697857	99.213	3.02	16.9	3.7
Rhizome length	LG4	chr04_45597769	127.674	3.12	17.4	3.7
Rhizome length	LG5	chr05_2131072	45.917	4.3	23.2	3.5
Rhizome length	LG5	chr05_3552366	46.626	5.41	28.3	3.2
Rhizome length	LG5	chr05_19559480	47.644	4.54	24.3	3.4
Rhizome length	LG8	chr08_9113282	56.893	3.41	18.9	3.7
Rhizome width	LG1	chr01_31131329	31.713	3.18	17.7	4.1
Rhizome width	LG1	chr01_31131332	31.713	3.24	18	4.1
Rhizome width	LG4	chr04_41435859	104.077	3.69	20.3	4.2
Rhizome width	LG4	chr04_18684136	104.707	3.52	19.4	4.2
Shoot canopy diameter	LG3	chr03_41784690	123.518	3.48	18.8	4.4
Shoot canopy diameter	LG4	chr04_50640951	117.62	3.06	16.7	4.5
Shoot canopy diameter	LG4	chr04_44959724	130.772	3.08	16.8	4.4
Shoot canopy diameter	LG6	chr06_24417495	37.652	3.33	18	4.3
Shoot canopy diameter	LG6	chr06_24417492	38.385	3.45	18.7	4.3
Shoot canopy diameter	LG6	chr06_8922282	38.939	3.45	18.7	4.1
Shoot canopy diameter	LG7	chr07_19256232	109.977	3.24	17.6	4.1
Shoot canopy diameter	LG8	chr08_16836275	60.222	3.58	19.3	4.2
Shoot canopy diameter	LG8	chr08_45317023	79.991	5.03	26	4.5
Shoot canopy diameter	LG8	chr08_8707992	80.031	3.36	18.2	4.4
Basal plant diameter	LG1	chr01_9917547	28.147	3.72	19.9	3.9
Basal plant diameter	LG2	chr02_4,417,521	84.524	3.24	17.6	4.1
Basal plant diameter	LG3	chr03_3184173	72.861	3.32	18	3.8
Basal plant diameter	LG4	chr04_23915754	86.789	5.35	27.4	4.3
Basal plant diameter	LG5	chr05_19136470	74.044	3.41	18.4	4.5
Basal plant diameter	LG5	chr05_19136429	76.756	3.18	17.3	4.3
Basal plant diameter	LG5	chr05_30967460	105.104	3.04	16.6	4.4
Basal plant diameter	LG8	chr08_8707992	80.031	3.32	18	4.4
Basal plant diameter	LG8	chr08_39307386	110.976	3.29	17.9	4.5
Stem number	LG2	chr02_11379759	84.524	4.21	22.3	4.3
Stem number	LG2	chr02_1782496	101.021	3.02	16.5	4.5
Stem number	LG4	chr04_1508899	62.807	3.07	16.8	4.5
Stem number	LG5	chr05_19136470	74.044	3.73	20	4.5
Stem number	LG7	chr07_1459167	98.102	3.56	19.2	3.9
Stem number	LG8	chr08_31605867	104.467	3.23	17.5	3.9
Absolute shoot height	LG6	chr06_22628780	118.818	3.71	19.9	4.5
Absolute shoot height	LG4	chr04_38,356,528	67.464	3.09	16.9	3.9
Absolute shoot height	LG7	chr07_34279538	92.741	3.08	16.8	4.3
Absolute shoot height	LG7	chr07_41091044	97.692	3.18	17.3	4.4
Absolute shoot height	LG8	chr08_42256225	141.879	3.87	20.7	4.3
Natural shoot height	LG1	chr01_49069297	150.782	3.54	19.1	4.4
Natural shoot height	LG1	chr01_49069365	152.757	3.57	19.2	4.4
Natural shoot height	LG2	chr02_21299826	63.993	3.46	18.7	4.5
Natural shoot height	LG2	chr02_30508990	78.382	3.83	20.5	4.1
Natural shoot height	LG2	chr02_38488687	121.387	3.02	16.5	4.3
Natural shoot height	LG2	chr02_38488685	121.475	3.02	16.5	4.3
Natural shoot height	LG2	chr02_7830781	134.138	3.83	20.5	4.4
Natural shoot height	LG2	chr02_38488729	135.291	3.88	20.7	4.3
Natural shoot height	LG3	chr03_27746993	93.877	4.07	21.6	4.1
Natural shoot height	LG4	chr04_36296510	87.645	3.26	17.7	4.3
Natural shoot height	LG5	chr05_39467311	57.638	3.5	18.9	4.3
Natural shoot height	LG5	chr05_19559628	64.596	3.33	18.1	4.5
Natural shoot height	LG5	chr05_20722800	120.884	3.04	16.6	4.0
Natural shoot height	LG5	chr05_20722779	120.884	3.03	16.6	4.0
Natural shoot height	LG6	chr06_32997819	67.553	4.67	24.4	4.3
Natural shoot height	LG7	chr07_7933121	63.778	3.03	16.6	4.5
Natural shoot height	LG7	chr07_5607731	85.279	3.39	18.4	4.3

**Figure 3:**
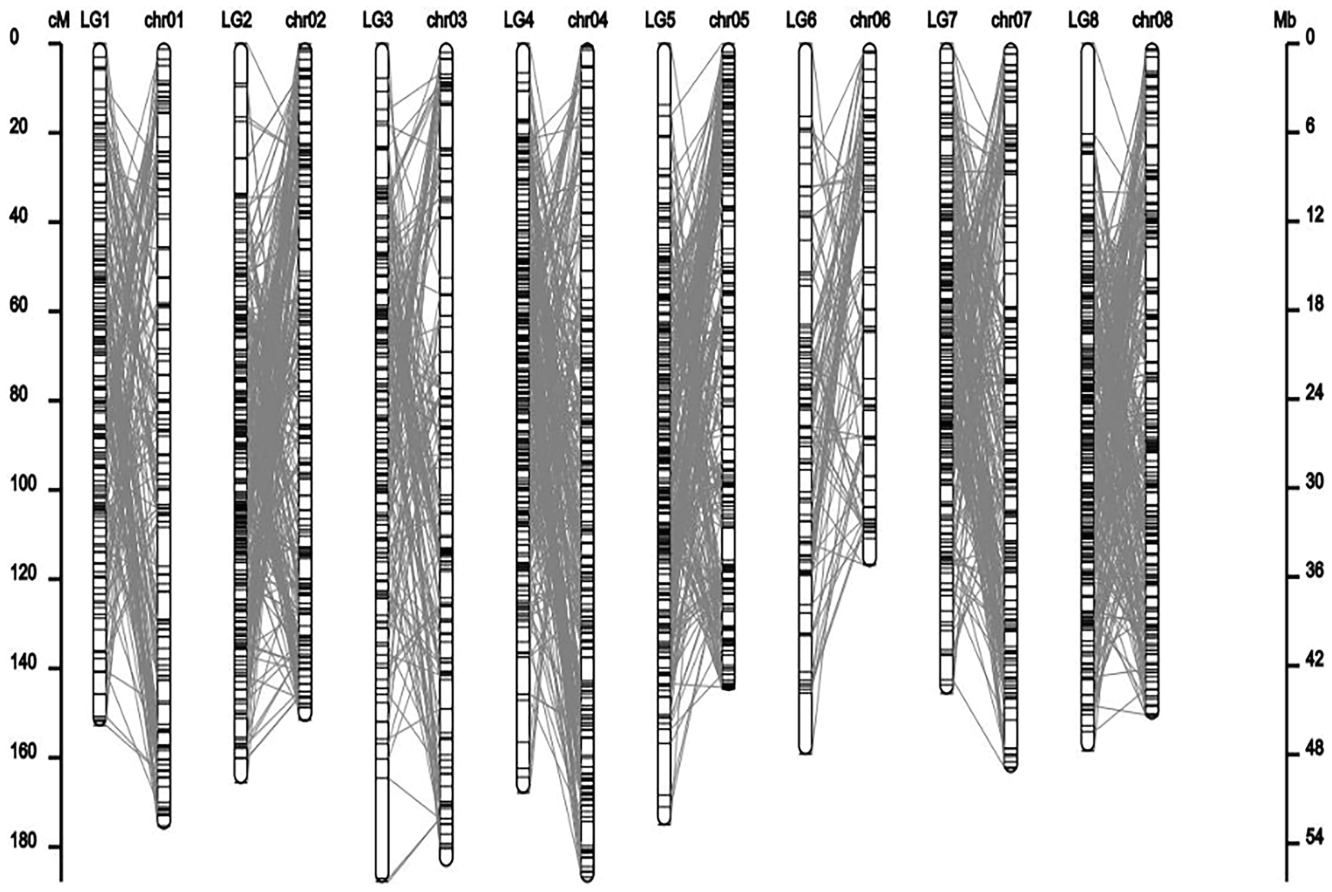
Collinearity analysis of genetic linkage map and physical map.

**Figure 4:**
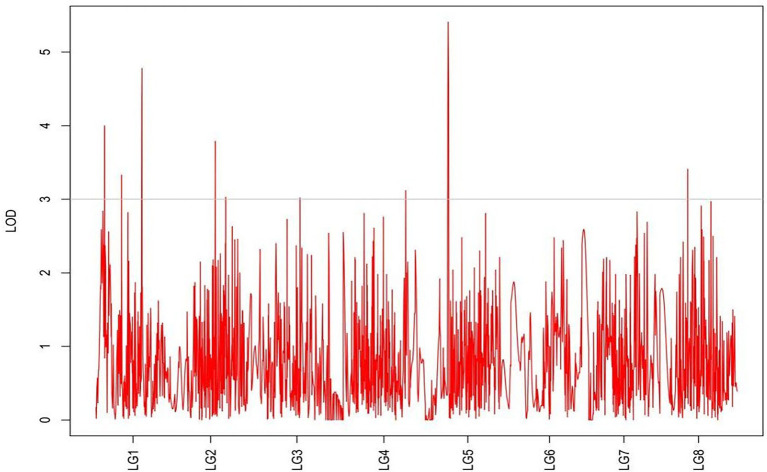
MapQTL localization of the rhizome length trait.

**Figure 5:**
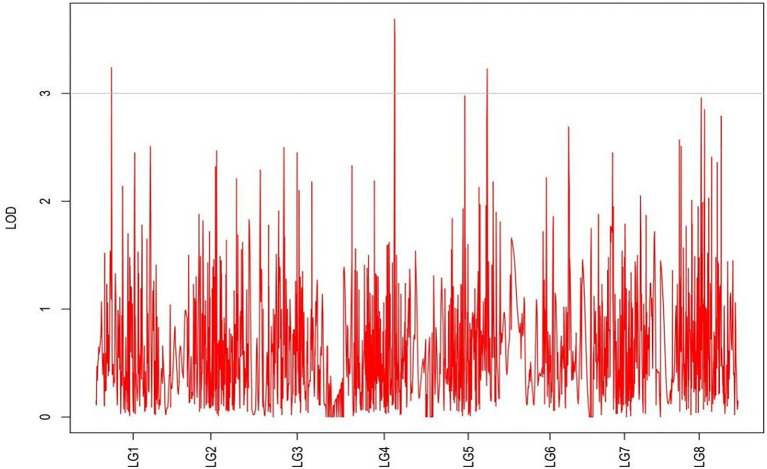
MapQTL localization of the rhizome width trait.

**Figure 6:**
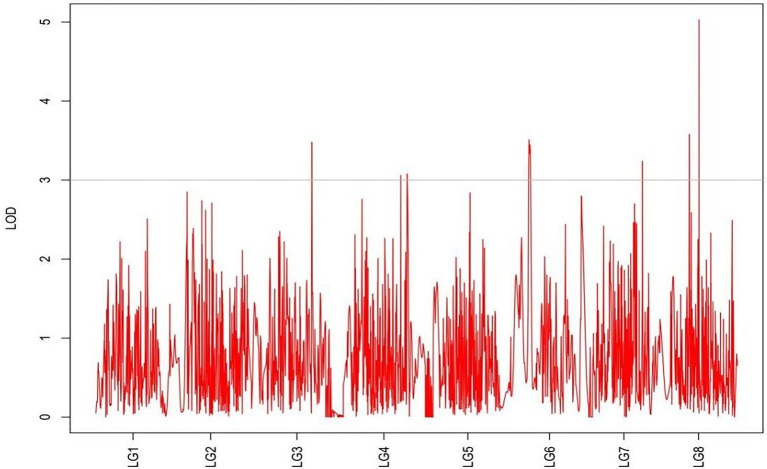
MapQTL localization of the shoot canopy diameter trait.

**Figure 7:**
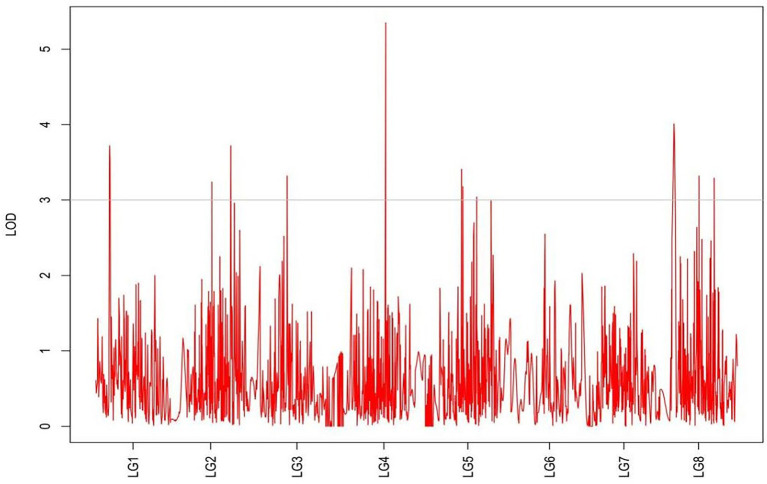
MapQTL localization of the basal plant diameter trait.

**Figure 8:**
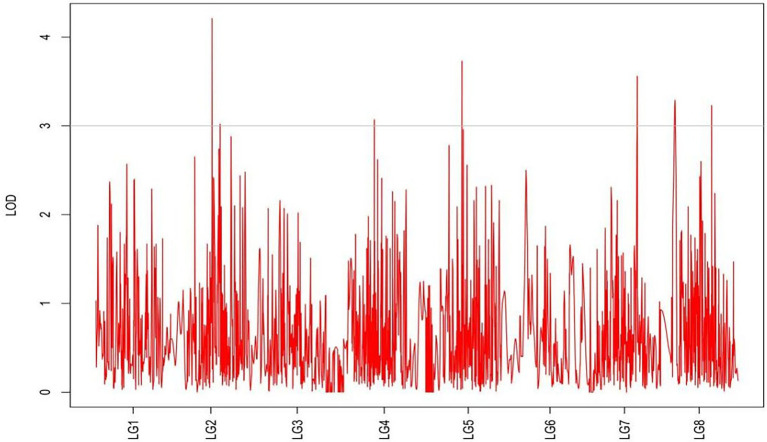
MapQTL localization of the stem number trait.

**Figure 9:**
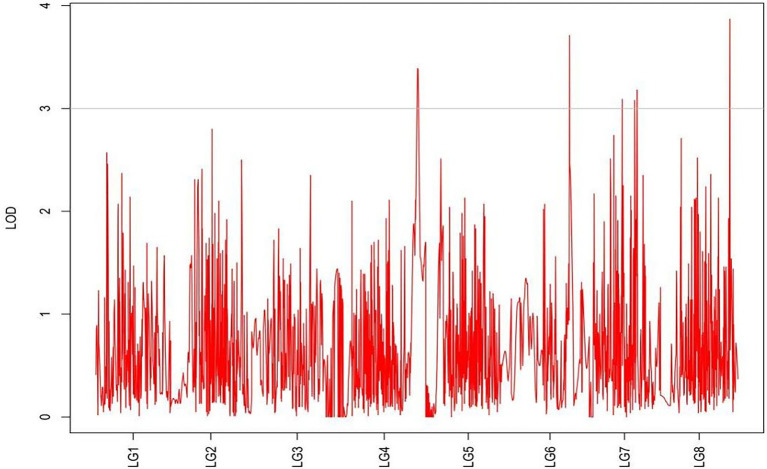
MapQTL localization of the absolute shoot height trait.

From the results in Table 5 and Figure 3, we identified 12 significant QTLs for the rhizome length in LG1, LG2, LG3, LG4, LG5, LG6, and LG8. The LOD values for the 12 QTLs were between 3.02 and 5.41 and the contribution ranged from 16.9% to 28.3%. The QTL with maximal LOD value was located in LG5, while the QTL with the minimal LOD was in LG3. Similarly, from the results in Table 6 and Figure 4, we identified four significant QTLs for the rhizome width in LG1 and LG4. The LOD values for the four QTLs were from 3.18 to 3.69 and the contribution ranged from 17.7 to 20.3%. From the results in Table 6 and Figure 5, we identified 10 significant QTLs for the shoot canopy diameter in LG3, LG4, LG6, LG7, and LG8. The LOD values for the QTLs were from 3.06 to 5.03 and the contribution ranged from 16.7% to 26%. From the results in Table 6 and Figure 6, we identified nine significant QTLs for the basal plant diameter in LG1, LG2, LG3, LG4, LG5, and LG8. The LOD values for the QTLs were from 3.04 to 5.35 and the contribution ranged from 16.6 to 27.4%. From the results in Table 6 and Figure 7, we identified 6 significant QTLs for the stem number in LG2, LG4, LG5, LG7, and LG8. The LOD values for the QTLs were from 3.02 to 4.21 and the contribution ranged from 16.5% to 22.3%. From the results in Table 6 and Figure 8, we identified five significant QTLs for the absolute shoot height inLG4, LG6, LG7, and LG8. The LOD values for the QTLs were from 3.08 to 3.87 and the contribution ranged from 16.8% to 20.7%. From the results in Table 6 and Figure 9, we identified 17 significant QTLs for the natural shoot height in LG1, LG2, LG3, LG4, LG5, LG6, and LG7. The LOD values for the QTLs were from 3.02 to 4.67 and the contribution ranged from 16.5% to 24.4%. The QTL with maximal LOD value was located in LG6 while the QTL with the minimal LOD was in LG2.

**Table 6:** Summary of genetic linkage map and distances.

Linkage group	SNPs number	Total genetic distance (cM)	Average genetic distance (cM)	Maximal gap (cM)
LG1	185	152.757	0.826	5.007
LG2	270	165.619	0.613	8.929
LG3	168	187.773	1.118	22.482
LG4	278	167.839	0.604	9.385
LG5	257	175.018	0.681	13.727
LG6	94	159.18	1.693	16.311
LG7	225	145.62	0.647	5.492
LG8	279	158.432	0.568	20.264
Total	1756	1312.238	0.844	22.482

**Figure 10:**
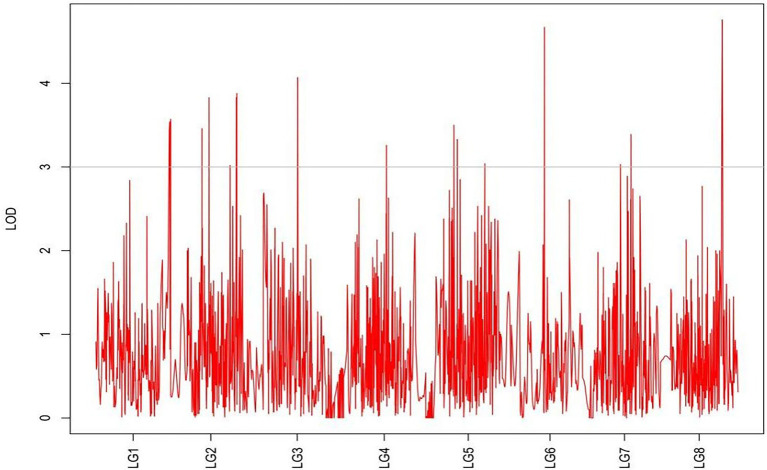
MapQTL localization of the natural shoot height trait.

For each trait, the corresponding QTL map was shown in Figures 4–10.

### Screening and identification of candidate genes for grazing tolerance

According to all the QTL ranges of grazing tolerance, we searched for genes in NCBI alfalfa genome database (CM001217.2), LG1 contained a total of 34 genes, LG2 obtained 35 genes, LG3 obtained 16 genes, LG4 found 30 genes, LG5 obtained 35 genes. Ten genes were obtained by LG6, 26 genes were obtained by LG7, and 29 genes were obtained by LG8. All the intervals contained 215 genes. Further analysis of gene annotation information was conducted to screen out 8 candidate genes that might be related to grazing tolerance, including genes related to MYB gene family, GRAS gene family, CAM gene family, etc. (Table 7).

**Table 7:** Screening results of candidate genes related to grazing tolerance.

LG	Gene ID	Gene description	Position (bp)	Character
LG4	LOC11422027	Scarecrow-like protein 14	23,915,589–23,918,205	Shoot canopy diameter
LG4	LOC11440209	Transcription factor TCP15	44,959,722–44,961,035	Shoot canopy diameter
LG8	LOC11429100	Gibberellin 20 oxidase 3	39,307,107–39,307,609	Basal plant diameter
LG7	LOC25498220	Ethylene-responsive transcription factor-like protein At4g13040	19,256,208–19,256,385	Shoot canopy diameter
LG4	LOC25493394	chlorophyll a-b binding protein AB80, chloroplastic	38,356,528–38,357,582	Absolute shoot height
LG6	LOC11414942	Calmodulin-7	8,920,707–8,928,256	Shoot canopy diameter
LG2	LOC25487134	Solanesyl diphosphate synthase 1	30,508,874–30,509,001	Natural shoot height
LG4	LOC11409053	Cell wall/vacuolar inhibitor of fructosidase 1	50,640,870–50,641,400	Shoot canopy diameter
LG6	LOC11412291	Transcription factor MYB30	3,615,590–3,617,827	Rhizome length
LG2	LOC11442911	Pathogenesis-related genes transcriptional activator PTI5	4,417,521–4,418,430	Basal plant diameter

The 10 candidate genes of *M. falcata* were verified by qRT-PCR. The results showed that the relative expression of LOC11422027 and LOC11429100 genes decreased continuously under different days of simulated cutting stress. The relative expression of LOC11442911 genes showed a trend of first increasing and then decreasing, and the relative expression of LOC25498220, LOC25493394, LOC11414942, LOC25487134, and LOC11409053 genes showed a trend of first decreasing and then increasing. The relative changes of LOC11412291 and LOC11440209 showed a trend of continuous up-regulation (Figure 10).

## Discussion

### RAD-seq analysis

Genetic map or genetic linkage map refers to the relative location and genetic distance of a gene or molecular marker on a chromosome, which is different from the real distance in a physical map (Meng et al., 2015; Yagi et al., 2017; Lv et al., 2021). Genetic distance in a genetic map is calculated from the recombination rate of genes or markers. For instance, 1% of recombination rate corresponds to approximately 1 cM (centimorgan). The farther two loci are located, the higher probability a recombination will occur or the higher the recombination rate. A recombination of 50% means the two loci are located in two different linkage groups. That is to say, the maximal value of a recombination rate is 50%. Normally, one chromosome is one linkage group. However, if a chromosome is very long, it is possible to consider different arms of a chromosome as different linkage groups. The genetic distance does not always correspond to a fixed physical distance. For example, in species with a high LD value, 1 cM corresponds to a slightly larger chromosomal fragment. Even in the same species, different chromosomal regions may be different. For instance, in the centromere region, 1 cM corresponds to a slightly larger chromosomal fragment (Chen, 2018). A high standard linkage map requires an average genetic distance of 20 cM for genetic markers on a chromosome. The distance between genetic markers for a QTL locus should be 10–20 cM or less (Zhang, 2020). The currently constructed genetic linkage maps for diploid and tetraploid alfalfa and the QTL marker analyses of important traits symbolize the successful application of genetic improvement and molecular breeding technologies in alfalfa (Musial et al., 2006, 2007a,b; Robins and Brummer, 2010; Han et al., 2011; Qiang et al., 2015).

**Figure 11:**
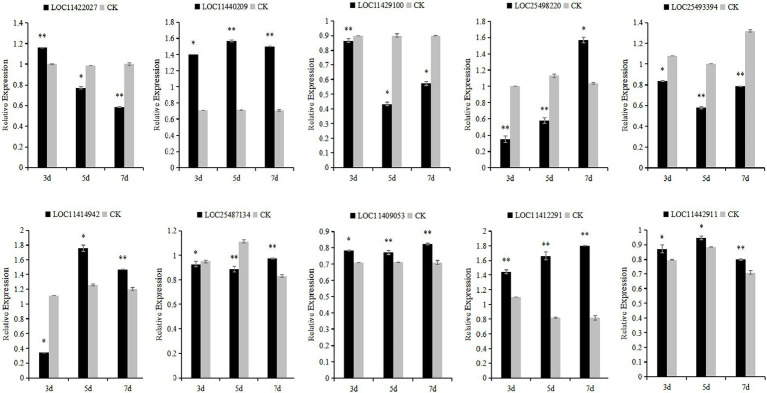
Relative expression levels of *Medicago falcata* after simulated cutting for 3, 5, and 7 days. Asterisks indicate significant differences as determined by ANOVA (^*^p < 0.05, ^**^p < 0.01).

In recent years, with the rapid development of molecular biology, different new molecular marker technologies have been developed. SNP markers are the third-generation molecular markers. Due to its high density, high representativeness, high genetic stability, and easy detection, SNPs markers are widely used in genetic map construction in various species (Han et al., 2012; Pandey et al., 2017a,b). The restriction-site-associated DNA sequencing (RAD-seq) technology is a high throughput sequencing approach, which greatly reduces the complexity of complex genomes and rapidly identifies genome-wide high-density SNPs (Feng et al., 2020). For species lacking reference genomes, RAD-seq overcomes the limitation of a known genome sequence yet obtains large scale of SNPs markers. Reducing the genome complexity means reducing cost, thus RAD-seq is especially useful in population-level studies. In classical SNP analysis, when SNPs are identified, researchers need to design specific primers to genotype individual samples. However, for RAD-seq, this genotypic information is acquired simultaneously with the identification of SNPs. For species with reference genome sequences, the analysis of RAD-seq is simple and novel SNPs can be identified. Therefore, RAD-seq is a new approach to develop thousands of SNPs markers with low cost. It has been applied in multiple model and non-model plant species. Recently, researchers in the United States performed RNA-seq in 27 tetraploid and diploid alfalfa genotypes. They identified 14,000 specific genes and 9 million SNPs and the construction of genetic linkage map and related QTL analysis are still in progress (Li et al., 2012, 2014; Fukuda et al., 2019; Wang L. et al., 2020). Using RAD-seq technology to detect SNP markers can obtain more polymorphic sites than Super GBS sequencing technology, and construct a higher density linkage map. Cui (2020) by using Super GBS sequencing technology, only obtained 460 SNP labeled in the Figure 11. Liu et al. (2017) obtained 4,346 SNP markers by RAD-sequence analysis. Wu et al. (2014) used RAD-seq technology to develop 3,804 pairs of new DNA markers, including SNPS and Indels, and combined with 1,230 SSR markers to construct a high-density genetic linkage map. Zhang et al. (2019) used the SNPs obtained by RAD-seq technology to construct a high-density genetic linkage map, including 4,346 SNP markers and 119 simple sequence repeat (SSR) markers. In this study, 79 individuals from the F1 population were used as the mapping population, and the RAD-seq technology was used for database construction and sequencing. To construct a high-density genetic map of alfalfa RAD-seq. Finally, a high-density genetic linkage map containing 8 linkage groups and 1,756 markers was obtained, with a total map distance of 1312.238 cM and an average density of 0.844 cM. The marker density of RAD-Seq map constructed by RAD-seq technology has been greatly improved, the resolution of QTL mapping has been improved, the length of QTL interval has been shortened, and the value of determining causal loci to improve the traits of interest has been improved. Therefore, RAD-seq technology was applied in this study to develop molecular markers for alfalfa mapping population.

In this study, the genetic linkage map and the physical map were drawn simultaneously using the progeny separation information and sequencing information. The average coverage distance of the genetic map was 164.03 cM, while the average coverage distance of the physical map was 46.43 Mb. It can be seen from the results that there are some differences between different mapping methods, and the positions of some markers on the genetic map and the physical map are inconsistent. Many plants, such as wheat and alfalfa, also have the phenomenon that the genetic and physical distances between markers on linkage groups are not consistent, mainly because the physical and genetic distances between markers in the repressed and active regions of chromosome recombination are not consistent (Zhao et al., 2017; Jiang et al., 2022). After the map construction, using bioinformatics analysis software for the above markers genome distribution of statistical genetic map and genome position corresponding to the relationship between genetic and physical location linear system such as high quality, and the accuracy of assessment to ensure map all indicators show that the research of *M. falcata* genetic linkage map construction with high quality and accuracy. These maps will be helpful for QTL mapping and marker-assisted selection (MAS) of alfalfa in the future.

### Grazing tolerance-associated QTL traits

QTLs are widely used in model plants and field crops, especially the important agronomic traits. QTL localization is to link specific traits with molecular makers on a genetic linkage map (Robins et al., 2007; Liu et al., 2014; Liu, 2016; Zhang, 2016). Using QTL mapping, some critical traits, such as yield (Wang L. et al., 2020) and cold tolerance (Chutimanitsakun et al., 2011; Shimoyama et al., 2020), have been linked to corresponding QTLs. Due to the practical values of QTL mapping in alfalfa, (Liu et al., 2017) located 19 QTL loci that are associated with agronomic traits in alfalfa. Zhang et al. (2020) detected 28 QTLs related to the important trait, flowering time. He (2016) constructed a genetic map and detected two QTLs associated with flowering time and leaf type in alfalfa. Tu (2011) identified 11 QTLs in *M. truncatula*. However, QTL analysis of agronomic traits in *M. falcata* is still in its preliminary stage. The success of QTL mapping in alfalfa and *M. truncatula* set a solid foundation for *M. falcata* studies.

In this study, we constructed a high-density genetic map in *M. falcata* and performed a QTL analysis for important grazing tolerance traits using interval mapping. Overall, we detected 63 QTLs for 6 grazing tolerance traits. These QTLs are distributed on eight LGs. Identification and location of the QTLs will aid in gene discovery, molecular marker-assisted selection, cloning, and regulation studies of quantitative trait-associated genes in *M. falcata*. These studies will eventually help improving and breeding the grazing tolerance varieties in *M. falcata*.

### Analysis of candidate genes for grazing tolerance traits

Previous QTL mapping studies on alfalfa mainly focused on flowering traits (Zhang et al., 2019, 2020). Although QTL mapping and RNA-seq integration have been applied to identify candidate genes in rice (*Oryza sativa* L.), maize (*Zea mays* L.), soybean (Glycine max), and other crops (Kuang et al., 2020; Lei et al., 2020; Han et al., 2022), but these methods have not been used to discover grazing tolerance trait genes in *M. falcata*.

In this study, 10 candidate genes that may be related to grazing tolerance were screened based on QTL mapping results, comparison of reference genomes, and functional annotation information. Among these candidate genes, LOC11422027 was located in Chr04.23915589–23918205. It is annotated as Scarecrow-like protein 14, which has been reported to be a multifunctional regulator involved in plant growth, photosynthesis, tolerance to photooxidative stress, and aging (Chen et al., 2014). The candidate gene LOC11429100 was located in LOC11429100 and was annotated as Gibberellin 20 oxidase 3, which is a key enzyme in GA biosynthesis (Yan et al., 2014). The candidate gene LOC25498220 was located in Chr07.19256208–19256385 and annotated as At4g13040 in Ethylene responsive transcription factor-like protein. At4g13040(referred herein as Apetala 2 family protein involved in SA-mediated disease defense 1—APD1) is an important regulator for SA-mediated plant defense (Mrunmay et al., 2014). In Chr04.38356528–38357582, LOC25493394 has an opposite pattern: A-B binding protein AB80, chloroplastic, (Gerardo et al., 1992) showed that chlorophyll A/B binding protein AB80 can promote chloroplast synthesis of coenzymes and improve the utilization of light energy in pea. The candidate gene LOC11414942 located in Chr06.8920707–8928256 was annotated as calmodulin-7, Recent studies further indicate that CAM7 is also an integral part of multiple signaling pathways including hormone, immunity, and stress (Zeb et al., 2019). Solanesyl diphosphate synthase 1 (SPS1) is the key enzyme in solanesol, a LOC25487134 gene located in Chr02.30508874–30509001 biosynthesis. Their studies in tobacco show that SPS1 significantly increased leaf growth, in tobacco, and in leaves content (Yan et al., 2020). LOC11409053 gene located in Chr04.50640870–50641400, Studies have shown that the Cell wall/vacuolar inhibitor of fructosidase 1 regulates ABA response and salt how in Arabidopsis (Yang et al., 2020). The candidate gene LOC11440209 located in Chr04.44959722–44961035 was annotated as the transcription factor TCP15, TCP15 are required for an efficient elongation response to auxin, most likely by regulating a subset of auxin-inducible genes related to cell expansion (Luciav et al., 2020). The candidate gene LOC11412291 was located in Chr06.3615590–3617827, which was annotated as the transcription factor MYB30. Some studies have reported that MYB30 is necessary for root growth regulation during defense responses and can regulate the synthesis of Arabidopsis wax powder. Epidermal wax powder plays an important role in plant resistance to diseases and insect pests and reduction of ultraviolet radiation (Raffaele et al., 2006; Kaho et al., 2018). Some studies have shown that the deeper the root is buried in the soil, the higher the grazing tolerance of alfalfa (Wang et al., 2013). The candidate gene LOC11442911 was located in Chr02.4417521-4418430 and was annotated as pathogen-related genes transcriptional activator PTI5. The ERF family transcription factor Pti5 belongs to a member of the ERF subfamily in the AP2/ERF family. Wang Y. et al. (2020) showed that Pti5 transcription factor plays a regulatory role in disease resistance and fruit ripening in tomato. qRT-PCR was used to analyze the relative expression levels of these 10 candidate genes through simulated grazing tolerance cutting test. We found that the relative expression levels of LOC11412291 and LOC11440209 were significantly up-regulated with the increase of cutting days. Therefore, we predicted that *M. falcata* faced with abiotic stress (cutting or grazing). LOC11412291 and LOC11440209 genes may have certain regulatory functions, which can be used as key candidate genes related to grazing tolerance.

## Conclusion

In this study, using RAD-seq technology, we sequenced 79 F1 individuals from a cross between a high (MF200401) and a low (MF200402) grazing tolerance *M. falcata* parent. After cleaning up reads and mapping to reference genome, we obtained 1756 high-quality SNPs and constructed a high-density genetic linkage map. These SNPs were located in 8 LGs with a consensus total length of 1312.238 cM and average distance of 0.844 cM between markers. Based on 6 phenotypic traits and linkage analysis, 63 QTLs associated with grazing tolerance traits were detected. Among them, 17 QTLs were associated with natural shoot height; 12 QTLs were related to rhizome length; 10 QTLs corresponded to shoot canopy diameter; 9 QTLs for basal plant diameter; 6 QTLs for stem number; 5 QTLs for absolute shoot height and 4 QTLs for rhizome width. Ten candidate genes that might be related to grazing tolerance were screened by QTL mapping and annotation information, and two key candidate genes (LOC11412291 and LOC11440209) were screened by qRT-PCR through simulated cutting test, and their functions could be further verified in *M. falcata*. The results presented in this study provide valuable information for breeding grazing tolerant alfalfa and *M. falcata*.

## Data availability statement

The data presented in the study are deposited in the NCBI repository, accession numbers: PRJNA795270 and PRJNA795570.

## Author contributions

XZho, XL, and DY assembled sequences and analyzed the data. XZho and XL wrote the manuscript. XZho collected the plant material. YZ and JW conceived the research and revised the manuscript. All authors contributed to the article and approved the submitted version.

## Funding

This work was supported by grants from the National Natural Science Foundation of China (no. 31560662 and 32160326) and Inner Mongolia Autonomous Region Science and Technology Project (no. 2020GG0176).

## Conflict of interest

The authors declare that the research was conducted in the absence of any commercial or financial relationships that could be construed as a potential conflict of interest.

## Publisher’s note

All claims expressed in this article are solely those of the authors and do not necessarily represent those of their affiliated organizations, or those of the publisher, the editors and the reviewers. Any product that may be evaluated in this article, or claim that may be made by its manufacturer, is not guaranteed or endorsed by the publisher.

## Footnotes

^1^ https://www.broadinstitute.org/GATK/
